# Microbial synthesis of propane by engineering valine pathway and aldehyde-deformylating oxygenase

**DOI:** 10.1186/s13068-016-0496-z

**Published:** 2016-04-01

**Authors:** Lei Zhang, Yajing Liang, Wei Wu, Xiaoming Tan, Xuefeng Lu

**Affiliations:** Key Laboratory of Biofuels, Shandong Provincial Key Laboratory of Synthetic Biology, Qingdao Institute of Bioenergy and Bioprocess Technology, Chinese Academy of Sciences, No. 189 Songling Road, Qingdao, 266101 China; Shandong Provincial Key Laboratory of Energy Genetics, Qingdao Institute of Bioenergy and Bioprocess Technology, Chinese Academy of Sciences, No. 189 Songling Road, Qingdao, 266101 China; University of Chinese Academy of Sciences, Beijing, China

**Keywords:** Propane, Valine pathway, Aldehyde-deformylating oxygenase, Synthetic biology, *Escherichia coli*

## Abstract

**Background:**

Propane, a major component of liquid petroleum gas (LPG) derived from fossil fuels, has widespread applications in vehicles, cooking, and ambient heating. Given the concerns about fossil fuel depletion and carbon emission, exploiting alternative and renewable source of propane have become attractive. In this study, we report the construction of a novel propane biosynthetic pathway in *Escherichia coli*.

**Results:**

We constructed an aldehyde reductases (ALR)-deprived *E. coli* strain BW25113(DE3) Δ13 via genetic engineering, which produced sufficient isobutyraldehyde precursors and finally achieved de novo synthesis of propane (91 μg/L) by assembling the engineered valine pathway and cyanobacterial aldehyde-deformylating oxygenase (ADO). Additionally, after extensive screening of ADO mutants generated by engineering the active center to accommodate branched-chain isobutyraldehyde, we identified two ADO mutants (I127G, I127G/A48G) which exhibited higher catalytic activity for isobutyraldehyde and improved propane productivity by three times (267 μg/L).

**Conclusions:**

The propane biosynthetic pathway constructed here through the engineered valine pathway can produce abundant isobutyraldehyde for ADO and overcome the low availability of precursors in propane production. Furthermore, the rational design aiming at the ADO active center illustrates the plasticity and catalytic potential of ADO. These results together highlight the potential for developing a microbial biomanufacturing platform for propane.

**Electronic supplementary material:**

The online version of this article (doi:10.1186/s13068-016-0496-z) contains supplementary material, which is available to authorized users.

## Background

Propane has become a promising fuel due to its remarkable properties such as higher energy density and liquefaction point than hydrogen, cleaner combustion and less greenhouse gas emission than gasoline, and compatibility with existing engine systems and transportation infrastructures [1]. Thus, it has been widely used in vehicle engines, cooking, and ambient heating [2]. There are reports that about 20 million tons of propane are consumed per year by vehicles, and more than 14 million homes get heat and energy from propane [3, 4]. Besides, it is also used as environment-friendly refrigerant and aerosol propellant to replace ozone-depleting chlorofluorocarbons (CFCs) [5]. Traditionally, propane is produced as a byproduct of petroleum refining, crude oil extraction, or natural gas exploitation. Considering the issues of carbon emission reduction and fossil fuel depletion, developing alternative and sustainable propane supply has gained tremendous attentions [6–8].

**Fig. 1:**
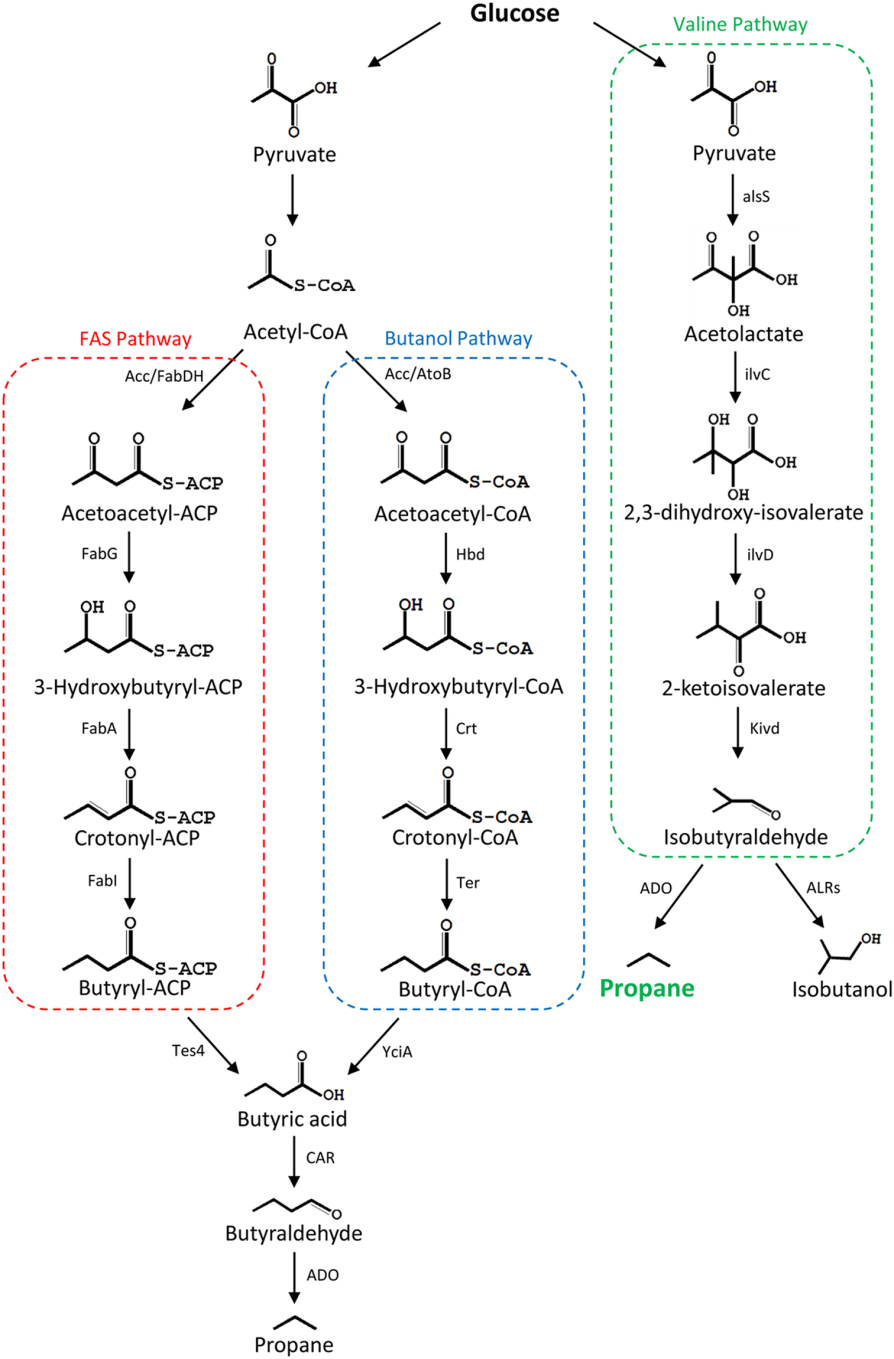
The comparison of different biosynthetic pathways for propane production. FAS pathway fatty acid biosynthesis pathway; Butanol pathway CoA-dependent butanol pathway of *Clostridium* sp.; *Acc* acetyl-CoA carboxylase, *FabDH* malonyl-CoA-ACP transacetylase and β-ketoacyl-ACP synthase, *FabG* β-ketoacyl-ACP reductase, *FabA* β-hydroxyacyl-ACP dehydrase, *FabI* enoyl-ACP reductase, *Tes4* acyl-ACP thioesterase from *Bacteroides fragilis*, *AtoB* acetyl-CoA acetyltransferase, *Hbd* 3-hydroxybutyryl-CoA dehydrogenase, *Crt* 3-hydroxybutyryl-CoA dehydratase, *Ter* trans-2-enoyl-CoA reductase, *YciA* acyl-CoA thioester hydrolase, *CAR* carboxylic acid reductase, *ADO* aldehyde-deformylating oxygenase, *alsS* acetolactate synthase, *ilvC* ketol-acid reductoisomerase, *ilvD* dihydroxy-acid dehydratase, *Kivd* alpha-ketoisovalerate decarboxylase, *ALR* aldehyde reductase

Up until recently, the first propane biosynthetic pathway was constructed by combining native fatty acid biosynthesis (FASII) of *E. coli* with the cyanobacterial aldehyde-deformylating oxygenase (ADO) [7, 8]. ADO is a non-heme diiron oxygenase and can catalyze the conversion of C_n_ fatty aldehydes to formate and corresponding C_n-1_ alkanes [9]. Oxygen and auxiliary reducing system are needed for ADO-catalyzed reaction [7–9]. This pathway relies on a thioesterase (Tes4) specific for butyryl acyl carrier protein (ACP), which releases the butyric acid from FASII specifically [10]. The butyric acid is converted into butyraldehyde by carboxylic acid reductase (CAR) from *Mycobacterium marinum* and further directed toward propane synthesis (red part of Fig. 1). However, the authors concluded that the propane productivity is limited by the total flux through FASII. In order to bypass this limitation, they designed a series of modified butyraldehyde pathways based on CoA-dependent butanol pathway commonly found in *Clostridium* sp. [11]. The pathway with best performance contains acetyl-CoA acetyltransferase (AtoB) from *E. coli*, 3-hydroxybutyryl-CoA dehydrogenase (Hbd) and 3-hydroxybutyryl-CoA dehydratase (Crt) from *Clostridium* sp., NADH-dependent trans-enoyl-CoA reductase (Ter) from *Treponema denticola*, thioesterase (YciA) from *Haemophilus influenza*, and CAR from *M. marinum*. These enzymes catalyze a series of reactions similar to those of the first propane pathway and convert acetyl-CoA into propane through CoA-dependent pathway instead of ACP-dependent FASII pathway (blue part of Fig. 1).

The valine pathway, one of the branched-chain amino acid (BCAA) pathways, might provide an alternative solution for precursor biosynthesis. Combined with the last two steps from Ehrlich pathway, its intermediate 2-ketosiovalerate can be converted into isobutyraldehyde by 2-keto-acid decarboxylase (KDC) and then converted into isobutanol by alcohol dehydrogenase (ADH) [12]. This pathway shows several advantages on biofuels production. First, it is well established on amino acid production technology and avoids the CoA-dependent pathway. Second, it has high isobutanol titer up to 22 g/L under micro-aerobic condition in *E. coli* [12]. Furthermore, it has shown broad feasibility in other promising microorganisms. For example, the pathway has been successfully constructed in *Synechococcus elongatus* PCC 7942, *Saccharomyces cerevisiae*, and *Bacillus subtilis* [13, 14]. By eliminating the last step that converts isobutyraldehyde into isobutanol, the high metabolites flux of valine pathway will end up with isobutyraldehyde which can be utilized as the substrate of ADO for propane production. Therefore, the valine pathway-based biosynthetic strategy might be an alternative and favorable choice for propane production.

In this study, we present a new biosynthetic pathway for propane based on the engineered valine pathway. By overcoming the insufficiency of precursors and enhancing the catalytic activity of ADO via rational design aiming at enzymatic substrate-access channel, we effectively demonstrate that the new pathway is one of promising solutions for microbial propane production.

## Results

### Biosynthesis of isobutyraldehyde, the precursor for propane production

First, an isobutyraldehyde synthetic pathway was constructed based on the valine pathway of *E. coli* (green part of Fig. 1) [15, 16]. The *alsS* gene from *B. subtilis* was introduced to convert pyruvate into 2-acetolactate, which is the first metabolic intermediate of valine pathway. For the second and third steps, the *ilvCD* genes of *E. coli* were overexpressed to convert 2-acetolactate into 2-ketoisovalerate. In the last two steps, *Kivd* from *Lactococcus lactis* transformed 2-ketoisovalerate into isobutyraldehyde, which could be converted into propane further by the *ADO* from *Prochlorococcus marinus* MIT 9313. All genes mentioned above were cloned into corresponding vectors, resulting plasmids pBAD33-alsS-Kivd, pAL96-ilvCD, and pET-PMT1231 (Additional file 1: Figure S1). To verify the feasibility of this pathway, we transformed those plasmids into *E. coli* BL21(DE3), anticipating fulfilling the biosynthesis of propane. However, there was no propane detected in the gas sample of headspace vial. Analyzing the culture components suggested that the isobutyraldehyde produced by valine pathway was reduced into isobutanol by endogenous ALRs of *E. coli*. It is well known that *E. coli* harbors many endogenous ALRs which transform aldehydes into alcohols, and they generate a great obstacle for alkane production [15, 16]. Therefore, it is essential to delete ALRs of *E. coli* host strain for propane production.

**Fig. 2:**
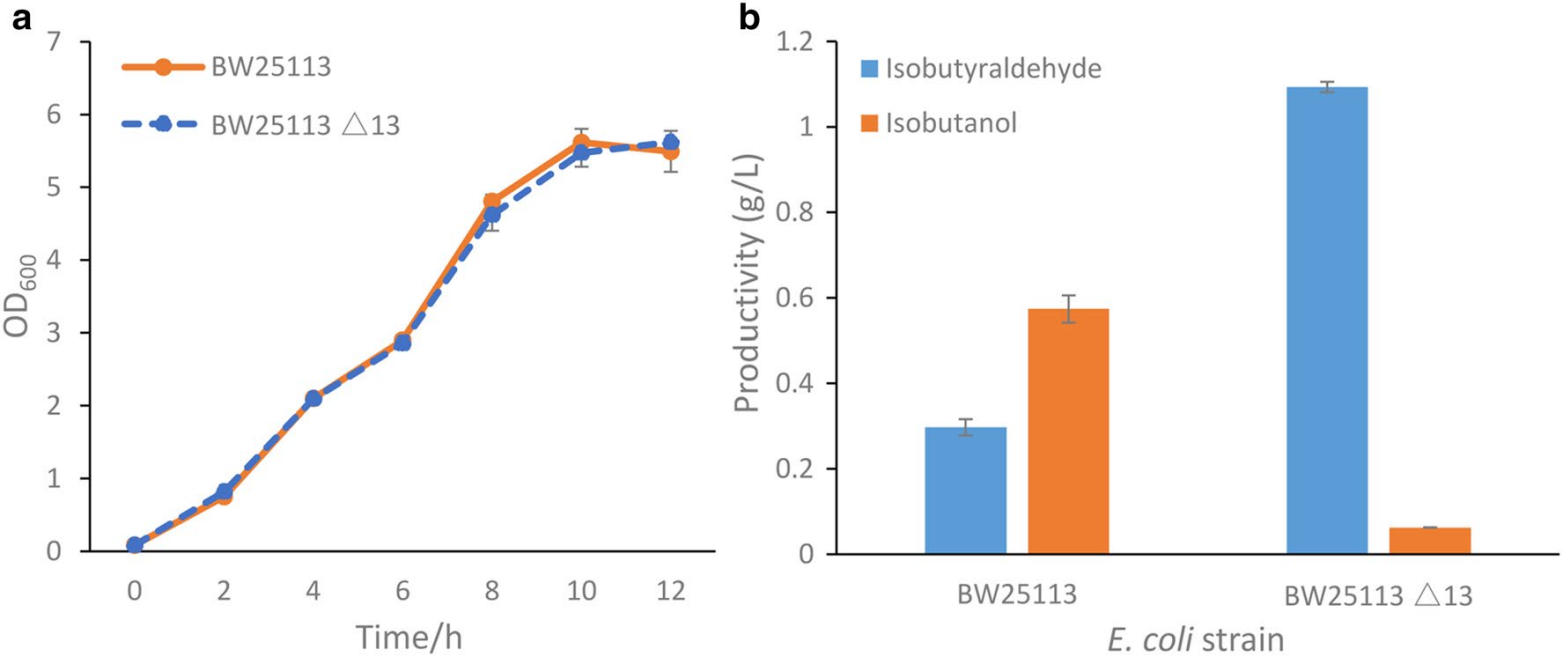
Biosynthesis of isobutyraldehyde by genetically engineered *E. coli* BW25113 Δ13. **a** Time-course of growth profiles of BW25113 and BW25113 Δ13. **b** Comparison of isobutyraldehyde and isobutanol production between two strains, BW25113 and BW25113 Δ13. A detailed protocol is provided in “Methods” section

A series of rationally targeted deletions of ALRs were carried out in *E. coli* BW25113, which had been proven well amenable for genetic engineering [17]. A two-step markerless recombination method was applied in this work [18, 19]. In light of the diverse specificity of ALRs on isobutyraldehyde and the functions that ALRs undertake in the metabolism of *E. coli*, we deleted 9 ALR genes, namely *yqhD*, *adhE*, *adhP*, *eutG*, *yiaY*, *yjgB*, *fucO*, *yahK*, and *DkgA,* following the rule that the deletions can decrease the ALR activity of *E. coli* and meanwhile do not inhibit cellular growth. The genetically engineered strain was verified by sequencing and named as BW25113 Δ13 (Additional file 1: Figure S2; Table S3). Given that 13 genes were deleted from the genome and some of those genes might have multiple functions, we inspected whether the strain could grow normally. The result indicated that the deletions did not hamper its growth (Fig. 2a). Although these gene deletions have no effect on cell growth, it is important to realize possible metabolic unbalance caused by the genetic manipulations here. For example, the *fnr* gene controls the transition between anaerobic and aerobic respiration by regulating a gene network which includes *adhE*, *frdABCD*, *ldhA*, and *pflB* [20]. In anaerobic condition, they use respective substrates as the terminal electron acceptors for oxidative phosphorylation, maintaining redox balance, and cellular growth [21]. The detailed examinations of these gene deletions on the physiological metabolism of the mutant strain are needed in the future study. We then evaluated the ability of BW25113 Δ13 to produce and accumulate isobutyraldehyde. Compared to the wild-type BW25113 which only accumulated 0.3 g/L isobutyraldehyde and 0.57 g/L isobutanol in 20 h, the engineered strain BW25113 Δ13 achieved an isobutyraldehyde titer up to 1.1 g/L, while very low amount of isobutanol was detected, indicating that 95 % activity of *E. coli* ALRs was eliminated (Fig. 2b). The results demonstrated that an *E. coli* strain nearly deprived of ALR activity had been successfully constructed, which could accumulate isobutyraldehyde as the major fermentation product.

### Conversion of isobutyraldehyde into propane

**Fig. 3:**
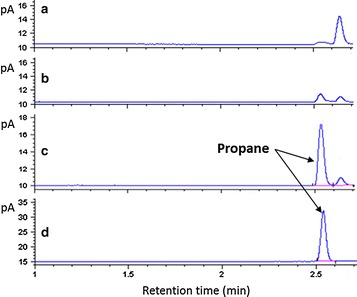
De novo biosynthesis of propane in *E. coli*. GC trace of gas sample from headspace vial is shown. **a** Wild-type BW25113. **b** BW25113(DE3) Δ13 expressing *alsS*, *ilvCD*, and *Kivd*. **c** BW25113(DE3) Δ13 expressing *alsS*, *ilvCD*, *Kivd*, and *ADO*. **d** Propane standard

In order to overexpress ADO with the pET system in the isobutyraldehyde-producing strain, the mutant strain BW25113 Δ13 was lysogenized by λDE3 lysogenization kit to build strain BW25113(DE3) Δ13. The plasmids pBAD33-alsS-Kivd, pAL96-ilvCD, and pET-PMT1231 were co-transformed into BW25113(DE3) Δ13, resulting in the strain BW25113(DE3) Δ13/Propane with an intact pathway for propane production (Additional file 1: Table S3). The strain was cultivated in TB media (30 g glucose/L) and induced with l-arabinose and isopropyl β-d-thiogalactopyranoside (IPTG) to produce propane. Finally, the propane was determined by gas chromatography (GC) (Fig. 3). The result showed that the strain BW25113(DE3) Δ13/Propane can successfully synthesize propane and no propane was formed in two control strains.

### Improvement of propane production by modifying ADO substrate specificity toward isobutyraldehyde

Although ADO is capable of catalyzing a wide range of aldehydes (C_3_–C_18_) to produce alka(e)nes, it exhibits low activity with the short-chain aldehydes (C_3_–C_5_) [7, 22, 23]. Given that the engineered valine pathway has produced abundant isobutyraldehyde and may achieve higher isobutyraldehyde titer after further metabolic optimizations, poor activity of ADO on isobutyraldehyde emerges as the major obstacle for higher propane productivity. To address this obstacle, we sought to enhance catalytic efficiency of ADO on isobutyraldehyde through rational design based on ADO structural analysis.

**Fig. 4:**
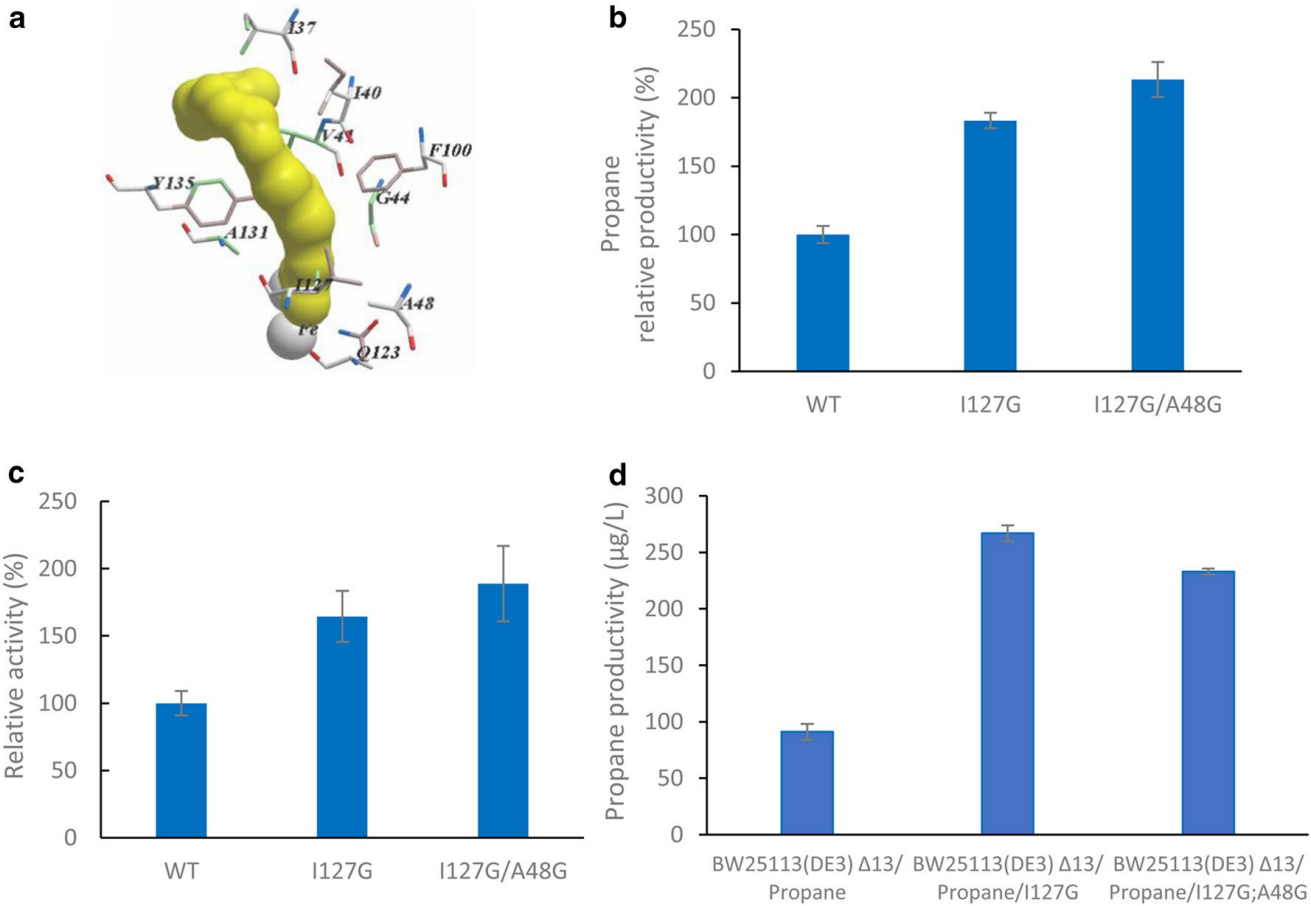
Improving propane production by rational design aiming at the active center of ADO. **a** Residues selected for mutagenesis. **b** Whole-cell assay of enzymes activity. Wild-type ADO is set at 100 %. A detailed protocol is provided in materials and methods. **c** In vitro enzymatic assay of mutants I127G, I127G/A48G. Wild-type ADO is set at 100 %. **d** De novo biosynthesis of propane using efficiency-enhanced mutants

Crystal structures of ADO (PMT1231) in complex with different substrates, some of which come from our previous studies, provide us an insight into substrate binding and catalytic mechanism of active center (Fig. 4a; Additional file 1: Figure S3) [9, 23–26]. We found that the substrate binding pocket of ADO capable of accommodating linear C_18_ aldehyde has too much redundant space for isobutyraldehyde (C_4_), while the width of substrate channel is relatively narrow for the branched methyl of isobutyraldehyde, especially near to the active center (Fig. 4a). It can be speculated that they are part of the reasons why ADO shows poor activity with the branched short-chain aldehydes. We therefore sought to engineer the substrate channel of ADO. First, residues Ala134 and Val41 were mutated into Phe or Tyr, aiming at that the introduced residues could shrink redundant space at the end of substrate binding pocket to fit the short chain (Fig. 4a; Additional file 1: Figure S3). Second, residues Ile127, Ala48, Ala131, Tyr135, Gln123, Gln123, Phe100, Ile40, and Ile37 were mutated into Gly or Ala to broaden substrate channel to accommodate the branched group (Fig. 4a; Additional file 1: Figure S3).

A whole-cell biotransformation method was used to assay the activity of the mutants [23]. Most mutants led to decline in propane production except the mutant I127G, which resulted in an 83 % increase in propane productivity compared to wild-type ADO (Fig. 4b; Additional file 1: Table S4). Based on this mutant, we subsequently investigated more double mutations (Additional file 1: Table S5). The mutants I127G/I37G and I127G/V41G increased upon propane productivity of I127G by 10.12 and 15.10 %, respectively. The propane productivity of the mutant I127G/A48G improved further and was more than two times greater than wild-type ADO (Fig. 4b; Additional file 1: Table S5). These results are corresponding with the in vitro enzymatic assay (Fig. 4c; Additional file 1: Table S6). Moreover, SDS-PAGE analysis showed that the mutant expression levels were consistent with wild-type ADO (Additional file 1: Figure S4).

We then introduced the mutations I127G and I127G/A48G into the strain BW25113(DE3) Δ13/Propane described above to investigate whether they could enhance de novo propane production. Compared to wild-type ADO, the pathway carrying mutant I127G and double mutant I127G/A48G yielded a 3-fold (267 μg/L) and 2.5-fold (233 μg/L) more propane, respectively (Fig. 4d). Although the double mutant I127G/A48G had better performance in whole-cell biotransformation and enzymatic assay experiments, it was the single mutant I127G that got highest propane productivity in de novo synthesis.

## Discussion

It is significant and promising to explore novel biosynthetic pathways producing renewable propane with higher efficiency. Among previous researches, butyraldehyde was used as the precursor of propane, which derived from FASII pathway of *E. coli* or CoA-dependent pathway from *Clostridium* sp. [8, 11]. Here we reported propane biosynthesis using isobutyraldehyde as the precursor based on the engineered valine pathway and the removal of ALR activity. The valine pathway possesses a very high metabolic flux proved by the reported isobutanol titer up to 22 g/L [12]. Considering the current isobutyraldehyde titer (1.1 g/L), it seems that the valine pathway still has 20-fold potential capacity to be exploited and implies sufficient precursor supply for propane production. To exploit the potential further, several metabolic optimizations are feasible. For example, more competitive genes such as *pta* (phosphotransacetylase gene) can be deleted to decrease the intermediates loss of valine pathway. Other optimizations such as deleting more *ALR* genes, increasing gene copy numbers of the pathway, eliminating the negative feedback inhibitions of BCAA pathways [27], as well as fed-batch fermentation, will also improve isobutyraldehyde and propane productivity. Given that the valine pathway has been successfully applied in several different model microorganisms described above, it could be applied widely to construct more different microbial platforms for propane biosynthesis [13, 14]. Beyond this, the ALRs-deprived *E. coli* strain constructed here can also serve as a microbial manufacturing host for other high value-added aromatic aldehydes which are precursors of flavors, fragrances, and pharmaceutical [28].

ADO natively catalyzes the conversion of long-chain aldehydes into corresponding alkanes [7, 22, 29–31]. To convert short-chain isobutyraldehyde into propane efficiently, the substrate specificity of ADO has to be modified for the utilization of the short-chain aldehydes. Some protein engineering works have been reported to alter natural specificity of ADO to convert short-chain aldehydes [23]. We tested those reported mutants in this study (data not shown here), but they failed to improve activity of ADO on isobutyraldehyde. Partially, it is because isobutyraldehyde is a shorter and branched aldehyde. ADO crystal structures in complex with different substrates have been solved and provide an insight into the substrate binding pocket, which is capable of accommodating up to 18-carbon-chain aldehyde and obviously too big for isobutyraldehyde. In addition, the substrate-access channel at the bottom of binding pocket is narrow and hampers the entrance of isobutyraldehyde due to the methyl group’s steric effect. In this work, we successfully improve the propane productivity through broadening substrate channel. Our works indicate that the structure-based rational design to broaden access channel and reshape the substrate binding pocket of ADO is effective. However, considering the isobutyraldehyde titer and potential of valine pathway, the activity of ADO variants still need improvement. Further engineering strategies include optimization of ADO binding pocket architecture and enhancement of ADO catalytic activity with release of H_2_O_2_ inhibition by fusing catalase or increase of electron supply by targeting reducing components such as NAD(P)H: flavodoxin/ferredoxin oxidoreductase [8, 31].

The highly active BCAA pathways are a powerful toolbox for biofuels production. Various types of 2-keto acids generated from BCAA pathways, such as 2-keto-3-methyl-valerate and 2-keto-4-methyl-pentanoate, can serve as precursors of the aldehydes, which can be further converted into the corresponding short-chain alkanes by ADO enzyme [12].

## Conclusion

We report a new biosynthetic pathway for propane production by assembling the engineered valine pathway of *E. coli* and cyanobacterial ADO. After enhancing the ADO activity via engineering the active center of ADO to accommodate branched-chain isobutyraldehyde, we significantly improved the propane production and effectively demonstrated that the new pathway is one of promising solutions for microbial propane production. However, to fulfill higher utilization ratio of isobutyraldehyde and greater propane productivity, more optimizations should be applied to improve the activity of ADO. It also provides a perspective to produce different types of short-chain alkanes by combination of the BCAA pathways and aldehyde-deformylating oxygenase.

## Methods

### Reagents

FastDigest restriction enzymes, pfu DNA polymerase, T_4_ DNA ligase, and media components were purchased from Thermo Scientific (Waltham, MA, USA). pEASY-Blunt Simple Cloning Kit was purchased from TransGen Biotech (Beijing, China). Plasmid miniprep kit, gel extraction kit, and PCR purification kit were purchased from Omega Bio-tek (Norcross, GA, USA). Gene sequencing and oligonucleotide synthesis were performed by Tsingke Biological Technology (Beijing, China). Isobutyraldehyde standard was purchased from Acros Organics (Geel, Belgium). All the other reagents were purchased from Sigma–Aldrich (St. Louis, Mo, USA).

### Strains and plasmids

#### Bw25113 Δ13

*Escherichia coli* K-12 BW25113 was used for genetic engineering and propane production. The *frdABCD*, *pflB*, *ldhA*, *fnr*, *yqhD*, *adhE*, *adhP*, *eutG*, *yiaY*, *yjgB*, *fucO*, *yahK*, and *DkgA* in BW25113 were deleted by a two-step markerless method to create the strain BW25113 Δ13. In this research, homology arms for genes deletion were constructed by fusion PCR. *EcoR*V restriction site was settled in the middle of homology arms. The Cat-sacB fragment could be cloned into the *EcoR*V restriction site and flanked with homology arms. All primers are listed in Additional file 1: Table S1.

#### pBAD33-alsS-Kivd

The *alsS* gene (GeneBank: 936852) from *B. subtilis* and *Kivd* gene (GeneBank: 8678808) from *L. lactis* were synthesized in Invitrogen (Shanghai, China) and cloned into pMD18-T vector, yielding pMD18-alsS and pMD18-Kivd, respectively. Primers alsS-SacI-F/alsS-fusion-R were used to amplify *alsS* fragment from pMD18-alsS. Primers Kivd-fusion-F/Kivd-SphI-R were used to amplify *Kivd* fragment from pMD18-Kivd. Then, fusion PCR was carried out to fuse *alsS* and *Kivd* fragments. The fragment, containing *alsS* and *Kivd* genes, was digested with *Sac*I and *Sph*I, cloned into pBAD33 plasmid, yielding pBAD33-alsS-Kivd. All primers are listed in Additional file 1: Table S2.

#### pAL96-ilvCD

The *ilvC* gene (GeneBank: 948296) and *ilvD* gene (GeneBank: 948277) were amplified from *E. coli* BW25113 genome with primers ilvC-SacI-F/ilvC-fusion-R and ilvD-fusion-F/ilvD-SalI-R. Then fusion PCR was carried out to fuse *ilvC* and *ilvD* fragments. The fragment, containing *ilvC* and *ilvD* genes, was digested with *Sac*I and *Sal*I, cloned into pAL96 plasmid, yielding pAL96-ilvCD. All primers are listed in Additional file 1: Table S2.

### pET-PMT1231 and pET-PMT1231 mutants

The codon-optimized *PMT1231* gene (Additional file 2) from *P*. *marinus* MIT 9313 was synthesized and cloned into pBluescript II SK(+) vector. The *Nde*I restriction site was introduced at 5′-terminal. Then, it was cloned into the pET-28a(+) vector at the restriction sites of *Nde*I and *Xho*I, yielding pET-PMT1231. PMT1231 mutants were created by site-directed mutagenesis and confirmed by sequencing. All primers are listed in Additional file 1: Table S2.

### Media, growth conditions, detections of isobutyraldehyde, isobutanol, and propane

*Escherichia coli* strains harboring recombinant plasmids were cultured in 3 mL Luria Broth (LB) liquid medium with appropriate antibiotics and incubated at 37 °C and 220 rpm overnight for inoculation.

To analyze and quantify isobutyraldehyde and isobutanol, 30 mL M9 minimal medium (30 g glucose/L) was inoculated with overnight culture at a 5 % (v/v) inoculation ratio and cultured at 37 °C and 220 rpm until OD_600_ reached 0.5. Then 30 mM l-arabinose and 0.3 mM IPTG were added to induce genes expression for 1 h. After induction, 10 mL of the culture was transformed into a 26 mL test tube that was tightly sealed to prevent evaporation of products. The test tube was further incubated for 20 h at 30 °C and 220 rpm. Then 500 μL supernatant of culture after centrifugation was extracted with same volume of toluene. 1 μL sample was injected into the GC equipped with a flame ionization detector (FID). The GC system is Agilent 7890A with a 7673A automatic injector which was held at 200 °C with a split ratio of 20:1. GC oven temperature was held at 63 °C for 5 min. The column is HP-INNOWAX (30 m × 0.32 mm × 0.25 μm film thickness, Agilent Technology). Nitrogen was used as the carrier gas at a flow of 1 mL/min. The FID temperature was maintained at 200 °C. Products peaks were identified by comparing with standards, and quantification was performed via calibration curve.

To analyze and quantify propane, 30 mL TB medium (12 g tryptone, 24 g yeast extract, 4 mL glycerol, 12.5 g K_2_HPO_4_, 2.3 g KH_2_PO_4_, and 30 g glucose per liter) was inoculated with overnight culture at a 5 % (v/v) inoculation ratio and cultured at 37 °C and 220 rpm until OD_600_ reached 0.5. Then, 30 mM l-arabinose and 0.3 mM IPTG were added to induce expression. The culture medium was further cultured for 3 h at 30 °C and 180 rpm. 1 mL of the culture was transferred into 8 mL tightly sealed vial. The vial was incubated for 1 h at 30 °C and 180 rpm. 200 μL gas sample of the headspace was injected manually into GC system with a Hamilton gas tight syringe. The method and setting of GC system were same as those described above. Propane peak was identified by comparing with standard, and quantification was performed via calibration curve.

### Whole-cell assay of enzymes activity

*Escherichia coli* BL21(DE3) harboring pET-PMT1231 mutations were cultured in 4 mL LB liquid media with kanamycin and incubated at 37 °C and 220 rpm overnight. 40 mL LB liquid medium was inoculated with overnight culture at a 5 % (v/v) inoculation ratio and further cultured until OD_600_ reached 0.9. Then 0.3 mM IPTG were added to induce ADO expression for 1 h. After induction, 10 mL of these culture was transformed into a 26 mL test tube. Isobutyraldehyde was added to the test tube at a 10 mM concentration. The tube was tightly sealed in time to prevent evaporation of isobutyraldehyde and propane. The tube was further incubated for 8 h at 30 °C and 220 rpm. 200 μL gas sample of the headspace was injected manually into GC system with a Hamilton gas tight syringe. The method and setting of GC system were same as those described above.

### Protein purification and in vitro enzymatic assay

The expression vectors pET-PMT1231, pET-PMT1231(I127G), and pET-PMT1231(I127G/A48G) were transformed into the competent *E. coli* BL21(DE3). Protein expression was carried out at 30 °C in super broth media. The cultures were induced with 0.25 mM IPTG when OD_600_ reached 0.6 and were shaken at 30 °C for additional 4 h. The cells harvested by centrifugation were disrupted by sonication. The proteins were purified with Nickel chelating resin, concentrated with Amicon YM10 membrane (10 kDa cut-off), and determined by the Bradford method [31]. Enzyme assay was carried out at 25 °C and used isobutyraldehyde as the substrate of ADO. Components of the reaction system are listed in Additional file 1: Table S6.

## Additional files

10.1186/s13068-016-0496-z Supplementary figures and tables.
10.1186/s13068-016-0496-z Codon-optimized gene sequence *PMT1231* from *P. marinus* MIT 9313.

## Abbreviations

ADH: alcohol dehydrogenase
ADO: aldehyde-deformylating oxygenase
ALR: aldehyde reductase
BCAA: branched-chain amino acid
CFC: chlorofluorocarbon
FASII: fatty acid biosynthesis
FID: flame ionization detector
GC: gas chromatography
IPTG: isopropyl β-d-thiogalactopyranoside
KDC: 2-keto-acid decarboxylase
LB: luria broth
LPG: liquid petroleum gas
OD_600_: optical cell density at 600 nm

## References

[1] Horng RF, Lai MP, Chang YP, Yur JP, Hsieh SF (2009). Plasma-assisted catalytic reforming of propane and an assessment of its applicability on vehicles. Int J Hydrogen Energ.

[2] Demçirbas A (2002). Fuel properties of hydrogen, liquefied petroleum gas (LPG), and compressed natural gas (CNG) for transportation. Energ Sources.

[3] Shelley C (2003). The story of LPG.

[4] Thorsteinsson HH, Tester JW (2010). Barriers and enablers to geothermal district heating system development in the United States. Energ Policy.

[5] Maione M, Giostra U, Arduini J, Furlani F, Graziosi F, Vullo EL (2013). Ten years of continuous observations of stratospheric ozone depleting gases at Monte Cimone (Italy)—comments on the effectiveness of the Montreal Protocol from a regional perspective. Sci Total Environ.

[6] Liu XY, Sheng J, Curtiss R (2011). Fatty acid production in genetically modified cyanobacteria. Proc Natl Acad Sci USA.

[7] Schirmer A, Rude MA, Li XZ, Popova E, del Cardayre SB (2010). Microbial biosynthesis of alkanes. Science.

[8] Kallio P, Pasztor A, Thiel K, Akhtar MK, Jones PR (2014). An engineered pathway for the biosynthesis of renewable propane. Nat Commun.

[9] Li N, Chang WC, Warui DM, Booker SJ, Krebs C, Bollinger JM (2012). Evidence for only oxygenative cleavage of aldehydes to alk(a/e)nes and formate by cyanobacterial aldehyde decarbonylases. Biochemistry.

[10] Jing FY, Cantu DC, Tvaruzkova J, Chipman JP, Nikolau BJ, Yandeau-Nelson MD (2011). Phylogenetic and experimental characterization of an acyl-ACP thioesterase family reveals significant diversity in enzymatic specificity and activity. BMC Biochem.

[11] Menon N, Pásztor A, Menon RK, Kallio P, Fisher K, Akhtar MK (2015). A microbial platform for renewable propane synthesis based on a fermentative butanol pathway. Biotechnol Biofuels.

[12] Atsumi S, Hanai T, Liao JC (2008). Non-fermentative pathways for synthesis of branched-chain higher alcohols as biofuels. Nature.

[13] Atsumi S, Higashide W, Liao JC (2009). Direct photosynthetic recycling of carbon dioxide to isobutyraldehyde. Nat Biotechnol.

[14] Jia X, Li S, Xie S, Wen J (2012). Engineering a metabolic pathway for isobutanol biosynthesis in *Bacillus subtilis*. Appl Biochem Biotechnol.

[15] Rodriguez GM, Atsumi S (2014). Toward aldehyde and alkane production by removing aldehyde reductase activity in *Escherichia coli*. Metab Eng.

[16] Rodriguez GM, Atsumi S (2012). Isobutyraldehyde production from *Escherichia coli* by removing aldehyde reductase activity. Microb Cell Fact.

[17] Datsenko KA, Wanner BL (2000). One-step inactivation of chromosomal genes in *Escherichia coli* K-12 using PCR products. Proc Natl Acad Sci USA.

[18] Jantama K, Zhang XL, Moore JC, Shanmugam KT, Svoronos SA, Ingram LO (2008). Eliminating side products and increasing succinate yields in engineered strains of *Escherichia coli*. Biotechnol Bioeng.

[19] Zhang XL, Jantama K, Moore JC, Shanmugam KT, Ingram LO (2007). Production of l-alanine by metabolically engineered *Escherichia coli*. Appl Environ Microbiol.

[20] Shalel-Levanon S, San KY, Bennett GN (2005). Effect of ArcA and FNR on the expression of genes related to the oxygen regulation and the glycolysis pathway in *Escherichia coli* under microaerobic growth conditions. Biotechnol Bioeng.

[21] Laouami S, Messaoudi K, Alberto F, Clavel T, Duport C (2011). Lactate dehydrogenase a promotes communication between carbohydrate catabolism and virulence in *Bacillus cereus*. J Bacteriol.

[22] Wang WH, Liu XF, Lu XF (2013). Engineering cyanobacteria to improve photosynthetic production of alka(e)nes. Biotechnol Biofuels.

[23] Khara B, Menon N, Levy C, Mansell D, Das D, Marsh ENG (2013). Production of propane and other short-chain alkanes by structure-based engineering of ligand specificity in aldehyde-deformylating oxygenase. ChemBioChem.

[24] Aukema KG, Makris TM, Stoian SA, Richman JE, Münck E, Lipscomb JD (2013). Cyanobacterial aldehyde deformylase oxygenation of aldehydes yields n-1 aldehydes and alcohols in addition to alkanes. ACS Catal.

[25] Krebs C, Bollinger JM, Booker SJ (2011). Cyanobacterial alkane biosynthesis further expands the catalytic repertoire of the ferritin-like “di-iron-carboxylate” proteins. Curr Opin Chem Biol.

[26] Jia C, Li M, Li J, Zhang JJ, Zhang HM, Cao P (2015). Structural insights into the catalytic mechanism of aldehyde-deformylating oxygenases. Protein Cell.

[27] Gusyatiner MM, Lunts MG, Koslov YI, Ivanovskaya LV, Voroshilova EB. DNA coding for mutant isopropylmalate synthase l-leucine producing microorganism and method for producing l-leucine. 2002. US Patent 6,403,342.

[28] Kunjapur AM, Tarasova Y, Prather KLJ (2014). Synthesis and accumulation of aromatic aldehydes in an engineered strain of *Escherichia coli*. J Am Chem Soc.

[29] Wu W, Zhang L, Yao L, Tan XM, Liu XF, Lu XF (2015). Genetically assembled fluorescent biosensor for in situ detection of bio-synthesized alkanes. Sci Rep.

[30] Coursolle D, Lian JZ, Shanklinb J, Zhao HM (2015). Production of long chain alcohols and alkanes upon coexpression of an acyl-ACP reductase and aldehyde deformylating oxygenase with a bacterial type-I fatty acid synthase in *E. coli*. Mol BioSyst.

[31] Zhang JJ, Lu XF, Li JJ (2013). Conversion of fatty aldehydes into alk (a/e)nes by in vitro reconstituted cyanobacterial aldehyde-deformylating oxygenase with the cognate electron transfer system. Biotechnol Biofuels.

[32] Tan ZG, Zhu XN, Chen J, Li QY, Zhang XL (2013). Activating phosphoenolpyruvate carboxylase and phosphoenolpyruvate carboxykinase in combination for improvement of succinate production. Appl Environ Microbiol.

