# Supplementary material for: Microbial synthesis of propane by engineering valine pathway and aldehyde-deformylating oxygenase
Source: Biotechnol Biofuels. 2016 Apr 1;9:80. doi: 10.1186/s13068-016-0496-z (PMC4818529; doi:10.1186/s13068-016-0496-z)
Supplement: Supplementary file 1 — 10.1186/s13068-016-0496-z Supplementary figures and tables. [file 13068_2016_496_MOESM1_ESM.docx]

**Supplementary Figures**


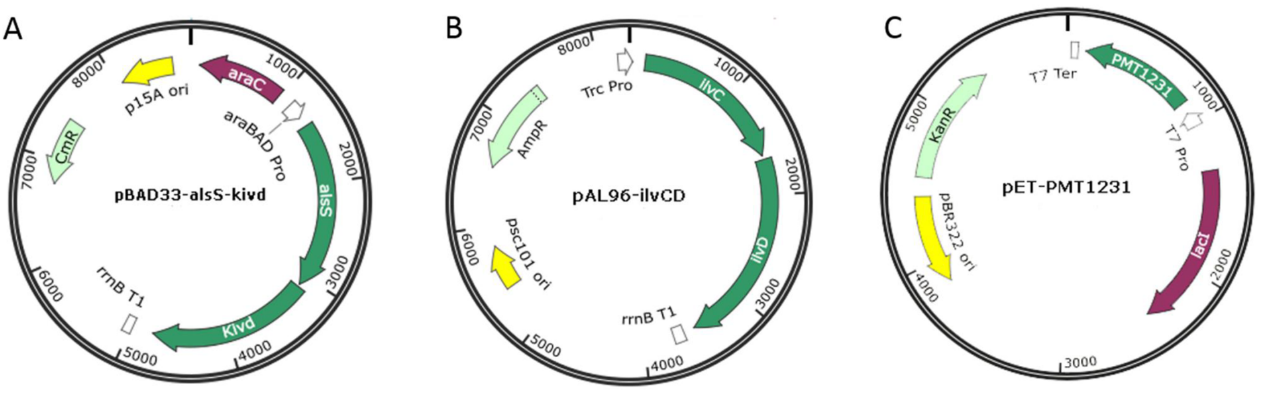


**Figure S1** The plasmids designed to construct propane synthetic pathway.


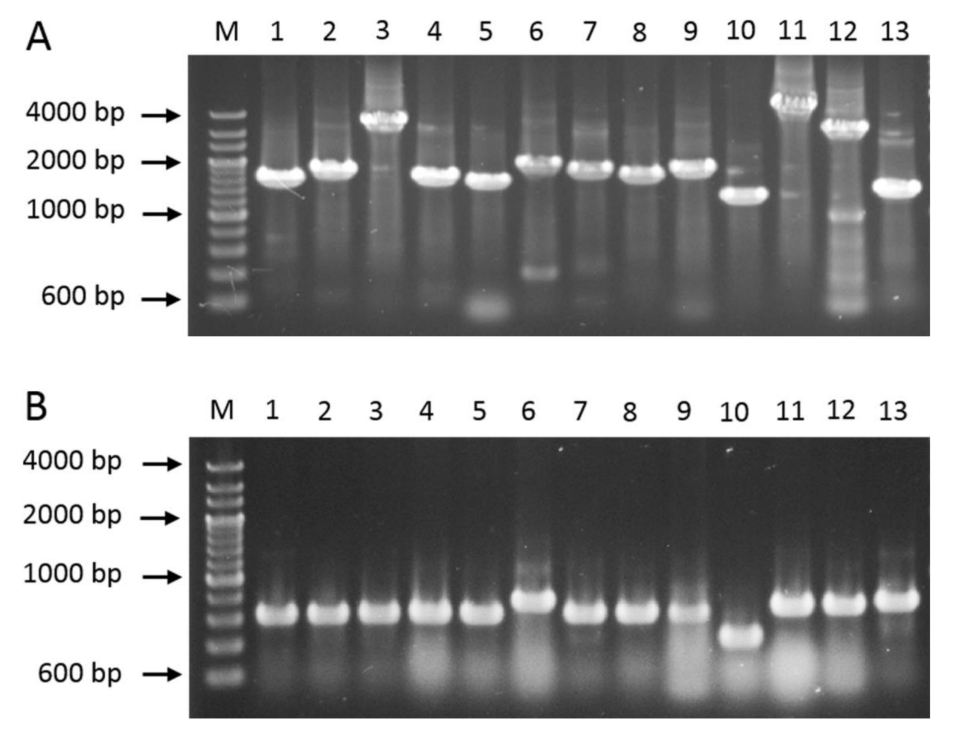


**Figure S2** PCR and agarose gel electrophoresis to verify genes deletion result. (**A**) PCR template is wild-type BW25113 genome. Lane M: Marker, Lane 1-13: *ldhA, yqhD, adhE, adhP, yjgB, eutG, yiaY, yahK, fucO, DkgA, frdABCD, pflB, fnr.* (**B**) PCR template is BW25113 △13 genome. Lane M: Marker, Lane 1-13: △*ldhA,* △*yqhD,* △*adhE,* △*adhP,* △ *yjgB,* △*eutG,* △*yiaY,* △*yahK,* △*fucO,* △*DkgA,* △frd*ABCD,* △*pflB,* △*fnr.*


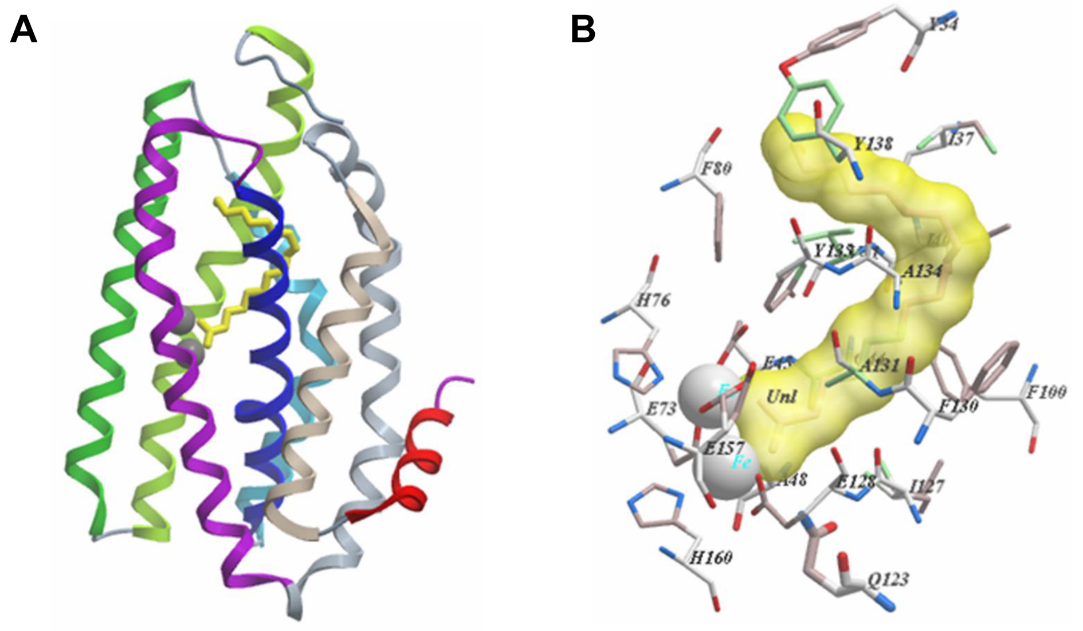


**Figure S3** (**A**) Overall structure of ADO (PDB ID 2OC5) from *P. marinus* MIT9313. Di-iron center is shown in grey, substrate is shown in yellow. (**B**) Details of the substrate, di-iron center, and key pocket-forming residues of ADO.


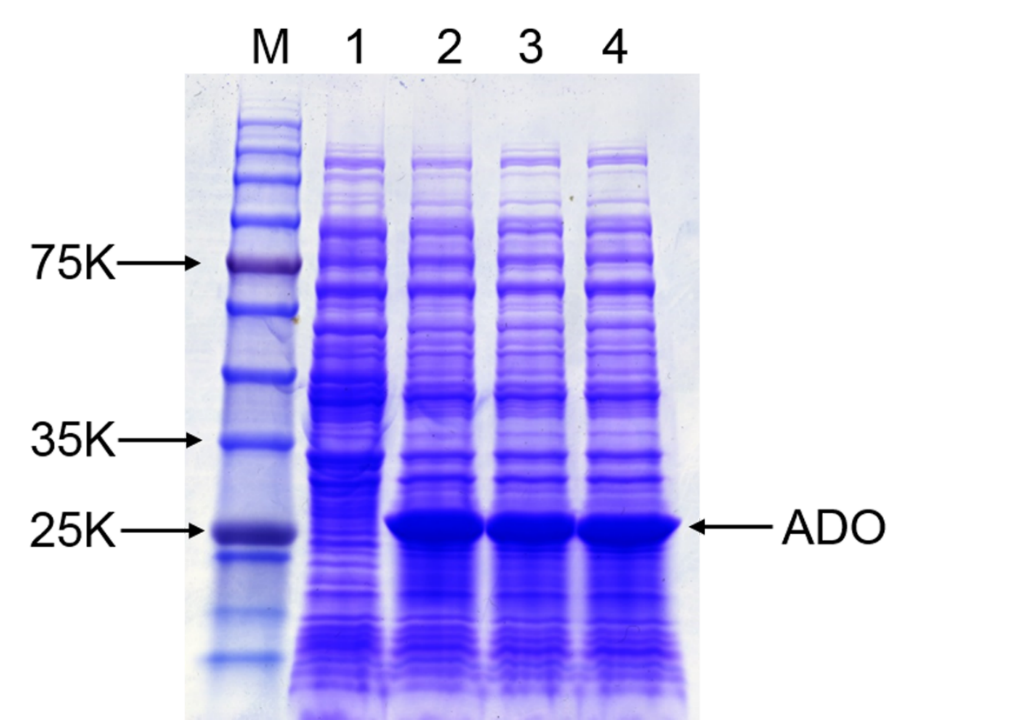


**Figure S4** SDS-PAGE analysis of express level of ADO mutants. Lane M: Marker, lane1: BL21(DE3) without expressing ADO, lane 2: BL21(DE3) expressing wild-type ADO, lane 3: BL21(DE3) expressing ADO(I127G), lane 4: BL21(DE3) expressing ADO(I127G/A48G).


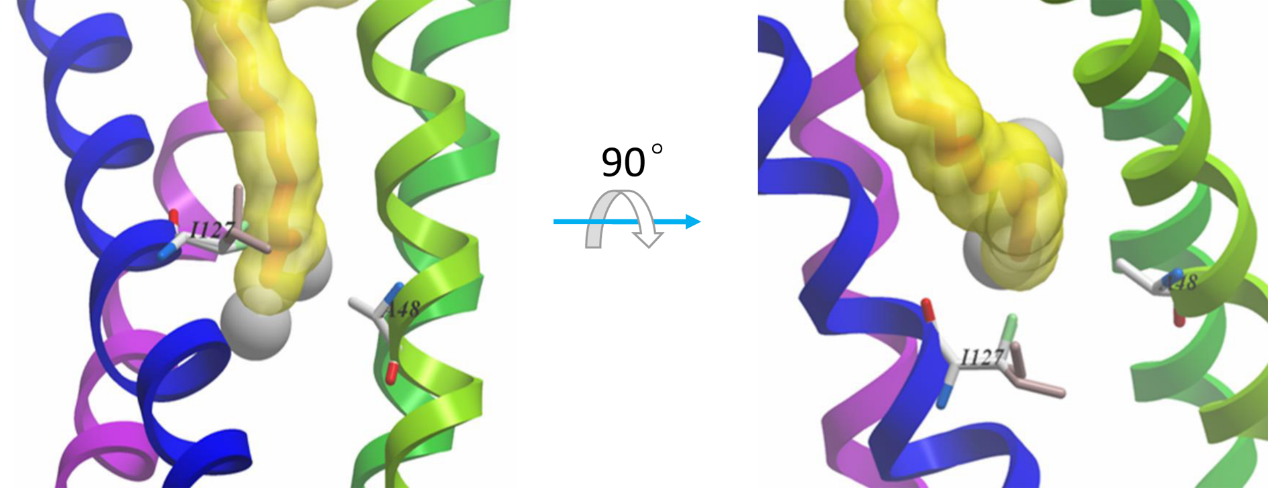


**Figure S5** Cut-away view of mutation sites I127 and A48 at two different angles.

**Supplementary Tables**

**Table S1** Primers used in strains construction

| Primer name | Primer sequence (5´- 3´) |
| --- | --- |
| yqhD-F | TCATATCGCGTAATTTCTTAGGAATAATG |
| yqhD-R | GATTACTTCCTCATTACTTGCTTGC |
| yqhD-fusion-F | GCAAAGGGAGCAAGTAATGGATATCTAAGCTTTTTACGCCTCAAAC |
| yqhD-fusion-R | TTGAGGCGTAAAAAGCTTAGATATCCATTACTTGCTCCCTTTGC |
| adhE-F | CTTGCTTACGCCACCTGGAAGTG |
| adhE-R | CAAATAGTTGTGCAGAGGGCGG |
| adhE-fusion-F | TTATCAGGAGAGCATTATGGATATCTAATCAGTAGCGCTGTCTGGCA |
| adhE-fusion-R | GCCAGACAGCGCTACTGATTAGATATCCATAATGCTCTCCTGATAATG |
| adhP-F | GCAACAGGCCATTGACGATAATTTCTG |
| adhP-R | CGTCCAGTGATTCCTGAATAATGTCCC |
| adhP-fusion-F | CATCCGAAAAGGAGGAACTGATATCGAGGCCTTTGCTGCGAC |
| adhP-fusion-R | GCAGCAAAGGCCTCGATATCAGTTCCTCCTTTTCGGATG |
| yjgB-F | TTCGGCACTGAAGAGGTATGCGGA |
| yjgB-R | AACCATGATATTGCCAATGTCAC |
| yjgB-fusion-F | CCAGAGAAGGACCAAAAAATGGATATCTGATCGAAAATTAACGACGCCAT |
| yjgB-fusion-R | ATGGCGTCGTTAATTTTCGATCAGATATCCATTTTTTGGTCCTTCTCTGG |
| eutG-F | CGCCCGGCATATTGAAGGGCA |
| eutG-R | GAACTGCGACAGGATCCTGTCTAC |
| eutG-fusion-F | AGGGGCTATATGCAAAATGAAGATATCGCGCAATAAATGCCGGATG |
| eutG-fusion-R | CATCCGGCATTTATTGCGCGATATCTTCATTTTGCATATAGCCCCT |
| yiaY-F | GAAAATCCTCAGTAAGCTGCCCG |
| yiaY-R | GACAGCTATCACGAATTTACGGGC |
| yiaY-fusion-F | CACTTTCAGGAGTGTGTTATGGATATCTAATCATCATTTCCACAACGGCT |
| yiaY-fusion-R | CGTTGTGGAAATGATGATTAGATATCCATAACACACTCCTGAAAGTG |
| yahK-F | GTCTTTTACAGATAGCAAATATCACACTTAC |
| yahK-R | CTGATTAAACCAGGCACTATCAGAAATCG |
| yahK-fusion-F | CATAGCTAATCAGGAGTAAACACAGATATCTGAAAAAATTAATAAATACCCTGTGG |
| yahK-fusion-R | CACAGGGTATTTATTAATTTTTTCAGATATCTGTGTTTACTCCTGATTAGCTATG |
| fucO-F | TCGCTTGTGAGGTGAATCTGG |
| fucO-R | AGAATCTGGATGCGGTTGCCGAAAAGT |
| fucO-fusion-F | ATTTCGTAAAGCAACAAGGAGAAGGGATATCTAAATGCGCTGATGTGATAATG |
| fucO-fusion-R | CATTATCACATCAGCGCATTTAGATATCCCTTCTCCTTGTTGCTTTACGAAAT |
| DkgA-F | TTTTACGCCTCAAACTTTCGTTTTCG |
| DkgA-R | CTGTTTTTCACCTCTCCGTTGCGT |
| DkgA-fusion-R | CACCGGGAGAATTTGCATGTTAGATATCACGTTCCTCCTTTATATGAACTCAC |
| DkgA-fusion-F | GTGAGTTCATATAAAGGAGGAACGTGATATCTAACATGCAAATTCTCCCGGTG |
| ldhA-F | TAATATCCTGATTTAGCGAAAAATTAAGC |
| ldhA-R | TAGCTGTTCTGGCGTAACAG |
| ldhA-fusion-F | CAACATCACTGGAGAAAGTCTTGATATCTCTTGCCGCTCCCCTGC |
| ldhA-fusion-R | GCAGGGGAGCGGCAAGAGATATCAAGACTTTCTCCAGTGATGTTG |
| frdABCD-F | GGTTTAGTAATTAAATTAATCATCTTCAGTG |
| frdABCD-R | ATAAATTACCGCCACCGCCATACC |
| frdABCD-fusion-F | GGATAAAAACAATCTGGAGGAATGTCGATATCACAATCTAACGCATCGCCAATGTA |
| frdABCD-fusion-R | TACATTGGCGATGCGTTAGATTGTGATATCGACATTCCTCCAGATTGTTTTTATCC |
| pflB-F | TTTTGGACCGCAGTCGGTTCTGC |
| pflB-R | CAAAGGAGTGAATGCGACCAATAAC |
| pflB-fusion-F | TTAAGAAGGTAGGTGTTACGATATCTAATTAGATTTGACTGAAATCGTACAGTAAAAAGC |
| pflB-fusion-R | CTGTACGATTTCAGTCAAATCTAATTAGATATCGTAACACCTACCTTCTTAAGTGGATTTT |
| fnr-F | CTGTAAACATTAAACAATTTGTGCCAGC |
| fnr-R | CCTGGTTAGGATCGATAACAACG |
| fnr-fusion-R | TGCGGAAAAATCAGATATCAGGTCTGCTCAAGCCGTAATTG |
| fnr-fusion-F | CGGCTTGAGCAGACCTGATATCTGATTTTTCCGCATAACTCAC |

**Table S2** Primers used in plasmids construction

| Primer name | Primer sequence (5´- 3´) |
| --- | --- |
| alsS-SacI-F | TTGGAGAGCTCGATAACAAGATACTGAGCACATCAGC |
| alsS-fusion-R | ATACATGGTACCTTTCTCCTCTTTAATGAACTAGAGAGCTTTCGTTTTCATGAGTTCC |
| Kivd-fusion-F | GGGGAACTCATGAAAACGAAAGCTCTCTAGTTCATTAAAGAGGAGAAAGGTACCATGTATACAGTAGG |
| Kivd-SphI-R | GGATTGCATGCTTATGATTTATTTTGTTCAGCAAATAGTTTG |
| ilvC-SacI-F | ACGCAGAGCTCACGAGGAATCACCATGGCTAACTACTT |
| ilvC-fusion-R | GTACTTAGGCATGGTATATCTCCTTCCGGGTGAGGGCATCAGCGC |
| ilvD-fusion-F | CCCTCACCCGGAAGGAGATATACCATGCCTAAGTACCGTTCCGCCACCA |
| ilvD-SalI-R | CGAGCGTCGACTTAACCCCCCAGTTTCGATTTATCG |
| A131F-F | TTGAGGCCTTTtttATCAGCGCTTACC |
| A131F-R | GGTAAGCGCTGATaaaAAAGGCCTCAA |
| G44F-F | ATCGTTATTGAAtttGAACAGGAAGCGCATG |
| G44F-R | TGCGCTTCCTGTTCaaaTTCAATAACGAT |
| A48F-F | GGTGAACAGGAAtttCATGACAATTACATTGCTA |
| A48F-R | TGTAATTGTCATGCGCTTCaaaTTCACCTTCAAT |
| A134F-F | TGCGATCAGCtttTACCACACTTACAT |
| A134F-R | ATGTAAGTGTGGTAaaaGCTGATCGCA |
| I127G-F | GCTGATCCAGGCTCTGCTGggTGAGGCCTTTGCGATCAGC |
| I127G-R | GCTGATCGCAAAGGCCTCAccCAGCAGAGCCTGGATCAGC |
| A131G-F | CTGCTGATTGAGGCCTTTGgtATCAGCGCTTACCACACTTACA |
| A131G-R | AGTGTGGTAAGCGCTGATacCAAAGGCCTCAATCAGCAGAG |
| Y135L-F | CCTTTGCGATCAGCGCTctgCACACTTACATTCCGGTAAGCGAC |
| Y135L-R | GCTTACCGGAATGTAAGTGTGcagAGCGCTGATCGCAAAGGC |
| I37G-F | GCATATTCTCGTggCAACGCCATCGTTATTGAAGGTG |
| I37G-R | AACGATGGCGTTGccACGAGAATATGCGTCCTTGTAGC |
| I40G-F | GTATCAACGCCggCGTTATTGAAGGTGAACAGGAAGC |
| I40G-R | TTCACCTTCAATAACGccGGCGTTGATACGAGAATATG |
| A48G-F | GAACAGGAAGgtCATGACAATTACATTGCTATCGG |
| A48G-R | TGTAATTGTCATGacCTTCCTGTTCACCTTCAATAACG |
| V41G-F | GTATCAACGCCATCGgTATTGAAGGTGAACAGGAAGC |
| V41G-R | CTTCAATAcCGATGGCGTTGATACGAGAATATG |
| Q123A-F | CCTGCTGATCgctGCTCTGCTGggTGAGGCCTTTG |
| Q123A-R | CAGCAGAGCagcGATCAGCAGGCAGGTCGGC |
| Q123G-F | CCTGCTGATCggtGCTCTGCTGggTGAGGCCTTTG |
| Q123G-R | CAGCAGAGCaccGATCAGCAGGCAGGTCGGC |
| F100A-F | GTGAATTTgcaGCACCGCTGCGCGACA |
| F100A-R | CAGCGGTGCagcAAATTCACGTGCGAAATCCATGTC |
| N162S-F | ATACTCACCTGtcCTATGGCGAAGCGTGGC |
| N162S-R | TTCGCCATAGgaCAGGTGAGTATATTCGTCTTTCACTA |

**Table S3** Strains and plasmids used in this study

| Name | Characteristics | Source |
| --- | --- | --- |
| **Strains** |  |  |
| BL21(DE3) | *F¯ ompT hsdSB(rB¯mB¯) gal dcm* (DE3) | Novagen |
| BW25113 | *rrnB3* Δ*lacZ4787* *hsdR514* Δ(*araBAD*)*567* Δ(*rhaBAD*)*568 rph-1* | Keio Collection |
| BW25113 Δ13 | BW25113 Δ*yqhD* Δ*adhE* Δ*adhP* Δ*eutG* Δ*yiaY* Δ*yjgB* Δ*fucO* Δ*yahK* Δ*DkgA* Δ*frdABCD* Δ*pflB* Δ*ldhA* Δ*fnr* | This study |
| BW25113(DE3) Δ13 | λDE3 prophage lysogen of BW25113 Δ13, prepared with λDE3 Lysogenization Kit | This study |
| BW25113(DE3) Δ13/ Propane | BW25113(DE3) Δ13 bearing pBAD33-alsS-Kivd, pAL96-ilvCD, and pET-PMT1231 | This study |
| BW25113(DE3) Δ13/ Propane/I127G | BW25113(DE3) Δ13 bearing pBAD33-alsS-Kivd, pAL96-ilvCD, and pET-PMT1231(I127G) | This study |
| BW25113(DE3) Δ13/ Propane/I127G;A48G | BW25113(DE3) Δ13 bearing pBAD33-alsS-Kivd, pAL96-ilvCD, and pET-PMT1231(I127G;A48G) | This study |
|  |  |  |
| **Plasmids** |  |  |
| pKD46 | Red recombinase expression vector; Amp^R^ | [1] |
| pXZ-CS | pEASY-Blunt vector bearing *cat* gene of pACYC184 and *SacB* gene from *B. subtilis* | [2] |
| pBAD33-alsS-Kivd | pBAD33 bearing *alsS* gene from *B. subtilis* and *Kivd* gene from *L. lactis;* Cm^R^ | This study |
| pAL96-ilvCD | pAL96 bearing *ilvCD* from *E.coli;* Amp^R^ | This study |
| pET-PMT1231 | pET-28a bearing *PMT1231* from *P. marinus* MIT 9313*;* Km^R^ | This study |
| pET-PMT1231 (I127G) | pET-28a bearing ADO mutation *PMT1231*(I127G); Km^R^ | This study |
| pET-PMT1231 (I127G;A48G) | pET-28a bearing ADO mutation *PMT1231*(I127G;A48G); Km^R^ | This study |

**Table S4** Whole-cell assay of mutants activity 1**.** Wild-type PMT1231 was set at 100%.

| Mutants | Relative activity |
| --- | --- |
| WT | 100.00% |
| A131F | 83.32% |
| G44F | 90.58% |
| A48F | 91.36% |
| A134F | 99.74% |
| A48F/A134F | 82.45% |
| G44F/A134F | 90.55% |
| I127G | 183.15% |
| A131G | 96.11% |
| Y135L | 82.51% |

**Table S5** Whole-cell assay of mutants activity 2. Mutant PMT1231 (I127G) was set at 100%.

| Mutants | Relative activity |
| --- | --- |
| I127G | 100.00% |
| I127G/I37G | 110.12% |
| I127G/I40G | 80.75% |
| I127G/A48G | 116.41% |
| I127G/V41G | 115.10% |
| I127G/Q123A | 101.93% |
| I127G/A48G/Q123G | 108.00% |
| I127G/F100A | 90.94% |
| I127G/N162S | 78.06% |

**Table S6** *In vitro* enzymatic assay of mutants activity [3].

| Component of reaction system (500 μL) | Concentration |
| --- | --- |
| Phenazine methosulfate | 75 μM |
| 5X HEPES buffer (PH=7.2) | 100 μL |
| Isobutylaldehyde | 5 mM |
| Catalase | 1 mg/mL |
| NADH | 750 μM |
| ADO Enzyme | 10 μM |
| (NH_4_)_2_Fe(SO_4_)_2_ | 40 μM |

**Supplementary References**

1. Datsenko KA, Wanner BL. One-step inactivation of chromosomal genes in *Escherichia coli* K-12 using PCR products. Proc Natl Acad Sci USA. 2000;97(12):6640-5.
2. Tan ZG, Zhu XN, Chen J, Li QY, Zhang XL. Activating phosphoenolpyruvate carboxylase and phosphoenolpyruvate carboxykinase in combination for improvement of succinate production. Appl Environ Microbiol. 2013;79: 4838–4844.
3. Zhang JJ, Lu XF, Li JJ. Conversion of fatty aldehydes into alk (a/e)nes by in vitro reconstituted cyanobacterial aldehydedeformylating oxygenase with the cognate electron transfer system. Biotechnol Biofuels. 2013;6:86.
