# Supplementary material for: Microbial synthesis of propane by engineering valine pathway and aldehyde-deformylating oxygenase
Source: Biotechnol Biofuels. 2016 Apr 1;9:80. doi: 10.1186/s13068-016-0496-z (PMC4818529; doi:10.1186/s13068-016-0496-z)
Supplement: Supplementary file 2 — 10.1186/s13068-016-0496-z Codon-optimized gene sequence PMT1231 from P. marinus MIT 9313. [file 13068_2016_496_MOESM2_ESM.docx]

Codon-optimized gene sequence *PMT1231* from *P*. *marinus* MIT 9313：

ATGCCGACTCTGGAGATGCCTGTTGCGGCGGTTCTGGATTCCACTGTTGGCTCTTCTGAAGCGCTGCCGGACTTCACCTCCGACCGCTACAAGGACGCATATTCTCGTATCAACGCCATCGTTATTGAAGGTGAACAGGAAGCGCATGACAATTACATTGCTATCGGTACCCTGCTGCCAGATCACGTTGAGGAACTGAAACGCCTGGCCAAAATGGAAATGCGTCACAAGAAAGGTTTTACCGCTTGCGGCAAAAACCTGGGCGTGGAAGCTGACATGGATTTCGCACGTGAATTTTTCGCACCGCTGCGCGACAACTTCCAGACGGCTCTGGGTCAGGGTAAAACGCCGACCTGCCTGCTGATCCAGGCTCTGCTGATTGAGGCCTTTGCGATCAGCGCTTACCACACTTACATTCCGGTAAGCGACCCGTTCGCACGCAAAATCACCGAAGGTGTAGTGAAAGACGAATATACTCACCTGAACTATGGCGAAGCGTGGCTGAAAGCGAACCTGGAATCCTGTCGTGAAGAGCTGCTGGAAGCTAACCGTGAAAACCTGCCGCTGATCCGTCGTATGCTGGATCAAGTGGCGGGCGACGCAGCAGTCCTGCAGATGGATAAAGAAGATCTGATCGAAGATTTCCTGATTGCGTACCAGGAGAGCCTGACCGAGATCGGTTTCAATACCCGTGAAATCACCCGTATGGCCGCTGCCGCACTGGTCTCTTAA
